# KrakenUniq: confident and fast metagenomics classification using unique *k*-mer counts

**DOI:** 10.1186/s13059-018-1568-0

**Published:** 2018-11-16

**Authors:** F. P. Breitwieser, D. N. Baker, S. L. Salzberg

**Affiliations:** 1 Center for Computational Biology, McKusick-Nathans Institute of Genetic Medicine, Johns Hopkins School of Medicine, Baltimore, MD USA; 2 Department of Computer Science, Johns Hopkins University, Baltimore, MD USA; 3 Departments of Biomedical Engineering and Biostatistics, Johns Hopkins University, Baltimore, MD USA

**Keywords:** Metagenomics, Microbiome, Metagenomics classification, Pathogen detection, Infectious disease diagnosis

## Abstract

False-positive identifications are a significant problem in metagenomics classification. We present KrakenUniq, a novel metagenomics classifier that combines the fast *k*-mer-based classification of Kraken with an efficient algorithm for assessing the coverage of unique *k*-mers found in each species in a dataset. On various test datasets, KrakenUniq gives better recall and precision than other methods and effectively classifies and distinguishes pathogens with low abundance from false positives in infectious disease samples. By using the probabilistic cardinality estimator HyperLogLog, KrakenUniq runs as fast as Kraken and requires little additional memory. KrakenUniq is freely available at https://github.com/fbreitwieser/krakenuniq.

**Electronic supplementary material:**

The online version of this article (10.1186/s13059-018-1568-0) contains supplementary material, which is available to authorized users.

## Background

Metagenomics classifiers attempt to assign a taxonomic identity to each read in a dataset. Because metagenomics data often contain tens of millions of reads, classification is typically done using exact matching of short words of length *k* (*k*-mers) rather than alignment, which would be unacceptably slow. The results contain read classifications but not their aligned positions in the genomes (as reviewed by [1]). However, read counts can be deceiving. Sequence contamination of the samples—introduced from laboratory kits or the environment during sample extraction, handling, or sequencing—can yield high numbers of spurious identifications [2, 3]. Having only small amounts of input material can further compound the problem of contamination. When using sequencing for clinical diagnosis of infectious diseases, for example, less than 0.1% of the DNA may derive from microbes of interest [4, 5]. Additional spurious matches can result from low-complexity regions of genomes and from contamination in the database genomes themselves [6].

Such false-positive reads typically match only small portions of a genome, e.g., if a species’ genome contains a low-complexity region, and the only reads matching that species fall in this region, then the species was probably not present in the sample. Reads from microbes that are truly present should distribute relatively uniformly across the genome rather than being concentrated in one or a few locations. Genome alignment can reveal this information. However, alignment is resource intensive, requiring the construction of indexes for every genome and a relatively slow alignment step to compare all reads against those indexes. Some metagenomics methods do use coverage information to improve mapping or quantification accuracy, but these methods require results from much slower alignment methods as input [7]. Assembly-based methods also help to avoid false positives, but these are useful only for highly abundant species [8].

Here, we present KrakenUniq, a novel method that combines very fast *k*-mer-based classification with a fast *k*-mer cardinality estimation. KrakenUniq is based on the Kraken metagenomics classifier [9], to which it adds a method for counting the number of unique *k*-mers identified for each taxon using the efficient cardinality estimation algorithm HyperLogLog [10–12]. By counting how many of each genome’s unique *k*-mers are covered by reads, KrakenUniq can often discern false-positive from true-positive matches. Furthermore, KrakenUniq implements additional new features to improve metagenomics classification: (a) searches can be done against multiple databases hierarchically; (b) the taxonomy can be extended to include nodes for strains and plasmids, thus enabling their detection; and (c) the database build script allows the addition of > 100,000 viruses from the NCBI Viral Genome Resource [13]. KrakenUniq provides a superset of the information provided by Kraken while running equally fast or slightly faster and while using very little additional memory during classification.

## Results

**Fig. 1:**
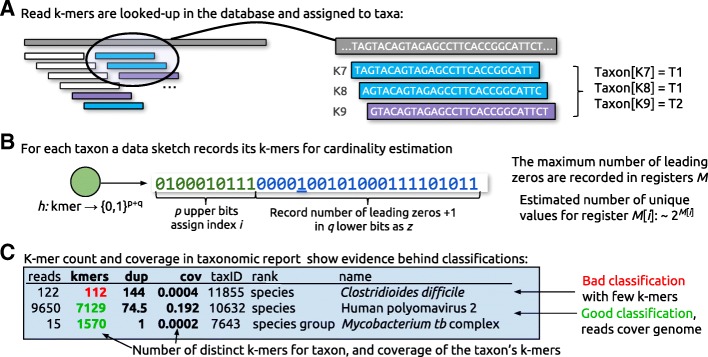
Overview of the KrakenUniq algorithm and output. **a** An input read is shown as a long gray rectangle, with *k*-mers shown as shorter rectangles below it. The taxon mappings for each *k*-mer are compared to the database, shown as larger rectangles on the right. For each taxon, a unique *k*-mer counter is instantiated, and the observed *k*-mers (K7, K8, and K9) are added to the counters. **b** Unique *k*-mer counting is implemented with the probabilistic estimation method HyperLogLog (HLL) using 16 KB of memory per counter, which limits the error in the cardinality estimate to 1% (see main text). **c** The output includes the number of reads, unique *k*-mers, duplicity (average time each *k*-mer has been seen), and coverage for each taxon observed in the input data

KrakenUniq was developed to provide efficient *k*-mer count information for all taxa identified in a metagenomics experiment. The main workflow is as follows: As reads are processed, each *k*-mer is assigned a taxon from the database (Fig. 1a). KrakenUniq instantiates a HyperLogLog data sketch for each taxon and adds the *k*-mers to it (Fig. 1b and Additional file 1: Section 1 on the HyperLogLog algorithm). After classification of a read, KrakenUniq traverses up the taxonomic tree and merges the estimators of each taxon with its parent. In its classification report, KrakenUniq includes the number of unique *k*-mers and the depth of *k*-mer coverage for each taxon that it observed in the input data (Fig. 1c).

### Efficient *k*-mer cardinality estimation using the HyperLogLog algorithm

**Fig. 2:**
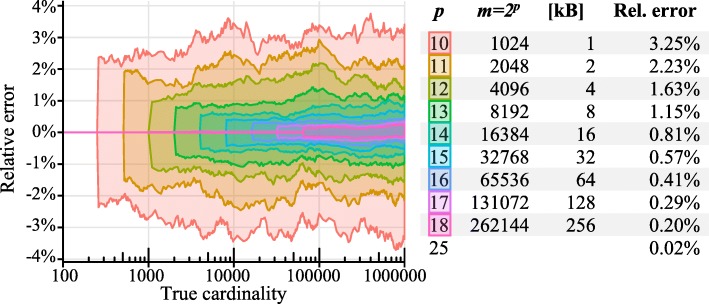
Cardinality estimation using HyperLogLog for randomly sampled *k*-mers from microbial genomes. Left: standard deviations of the relative errors of the estimate with precision *p* ranging from 10 to 18. No systematic biases are apparent, and, as expected, the errors decrease with higher values of *p*. Up to cardinalities of about 2^*p*^/4, the relative error is near zero. At higher cardinalities, the error boundaries stay near constant. Right: the size of the registers, space requirement, and expected relative error for HyperLogLog cardinality estimates with different values of *p*. For example, with a precision *p* = 14, the expected relative error is 0.81%, and the counter only requires 16 KB of space, which is three orders of magnitude less than that of an exact counter (at a cardinality of one million). Up to cardinalities of 2^*p*^/4, KrakenUniq uses a sparse representation of the counter with a higher precision of 25 and an effective relative error rate of about 0.02%

Cardinality is the number of elements in a set without duplicates, e.g., the number of distinct words in a text. An exact count can be kept by storing the elements in a sorted list or linear probing hash table, but that requires memory proportional to the number of unique elements. When an accurate estimate of the cardinality is sufficient, however, the computation can be done efficiently with a very small amount of fixed memory. The HyperLogLog algorithm (HLL) [10], which is well suited for *k*-mer counting [14], keeps a summary or *sketch* of the data that is sufficient for precise estimation of the cardinality and requires only a small amount of constant space to estimate cardinalities up to billions. The method centers on the idea that long runs of leading zeros, which can be efficiently computed using machine instructions, are unlikely in random bitstrings. For example, about every fourth bitstring in a random series should start with 01_2_ (one 0 bit before the first 1 bit), and about every 32nd hash starts with 00001_2_. Conversely, if we know the maximum number of leading zeros *k* of the members of a random set, we can use 2^*k* + 1^ as a crude estimate of its cardinality (more details in Additional file 1: Section 1 on the HLL algorithm). HLL keeps *m* = 2^*p*^ 1 byte counts of the maximum numbers of leading zeros on the data (its data *sketch*), with *p*, the precision parameter, typically between 10 and 18 (see Fig. 2). For cardinalities up to about *m*/4, we use the sparse representation of the registers suggested by Heule et al. [11] that has the much higher effective precision *p*′ of 25 by encoding each index and count in a vector of 4-byte values (see Fig. 2). To add a *k*-mer to its taxon’s sketch, the *k*-mer (with *k* up to 31) is first mapped by a hash function to a 64-bit hash value. Note that *k*-mers that contain non-A, C, G, or T characters (such as ambiguous IUPAC characters) are ignored by KrakenUniq. The first *p* bits of the hash value are used as index *i*, and the later 64-*p* = *q* bits for counting the number of leading zeros *k*. The value of the register *M*[*i*] in the sketch is updated if *k* is larger than the current value of *M*[*i*].

When the read classification is finished, the taxon sketches are aggregated up the taxonomy tree by taking the maximum of each register value. The resulting sketches are the same as if the *k*-mers were counted at their whole lineage from the beginning. KrakenUniq then computes cardinality estimates using the formula proposed by Ertl [12], which has theoretical and practical advantages and does not require empirical bias correction factors [10, 11]. In our tests, it performed better than Flajolet’s and Heule’s methods (Additional file 1: Figures S1 and S2).

The expected relative error of the final cardinality estimate is approximately 1.04/sqrt(2^*p*^) [10]. With *p* = 14, the sketch uses 2^14^ 1-byte registers, i.e., 16 KB of space, and gives estimates with relative errors of less than 1% (Fig. 2). Note that KrakenUniq also incorporates an exact counting mode, which however uses significantly more memory and runtime without appreciable improvements in classification accuracy (see the “Exact counting versus estimated cardinality” section).

### Results on 21 simulated and 10 biological test datasets

We assessed KrakenUniq’s performance on the 34 datasets compiled by McIntyre et al. [15] (see Additional file 2: Table S3 for details on the datasets). We place greater emphasis on the 11 biological datasets, which contain more realistic laboratory and environmental contamination. In the first part of this section, we show that unique *k*-mer counts provide higher classification accuracy than read counts, and in the second part, we compare KrakenUniq with the results of 11 metagenomics classifiers. We ran KrakenUniq on three databases: “orig,” the database used by McIntyre et al.; “std,” which contains all current complete bacterial, archaeal, and viral genomes from RefSeq plus viral neighbor sequences and the human reference genome; and “nt,” which contains all microbial sequences (including fungi and protists) in the non-redundant nucleotide collection nr/nt provided by NCBI (see Additional file 1: Section 2 for details). The “std” database furthermore includes the UniVec and EmVec sequence sets of synthetic constructs and vector sequences, and low-complexity *k*-mers in microbial sequences were masked using NCBI’s dustmasker with default settings. We use two metrics to compare how well methods can separate true positives and false positives: (a) F1 score, i.e., the harmonic mean of precision *p* and recall *r*, and (b) recall at a maximum false discovery rate (FDR) of 5%. For each method, we compute and select the ideal thresholds based on the read count, *k*-mer count or abundance calls. Precision *p* is defined as the number of correctly called species (or genera) divided by the number of all called species (or genera) at a given threshold. Recall *r* is the proportion of species (or genera) that are in the test dataset and that are called at a given threshold. Higher F1 scores indicate a better separation between true positives and false positives. Higher recall means that more true species can be recovered while controlling the false positives.

Because the NCBI taxonomy has been updated since the datasets were published, we manually updated the “truth” sets in several datasets (see Additional file 1: Section 2.3 for details on taxonomy fixes). Any cases that might have been missed would result in a lower apparent performance of KrakenUniq. Note that we exclude the over 10-year-old simulated datasets simHC, simMC, and simLC from Mavromatis et al. (2007), as well as the biological dataset JGI SRR033547 which has only 100 reads.

#### Classification performance using unique *k*-mer or read count thresholds

We first looked at the performance of the unique *k*-mer count thresholds versus read count thresholds (as would be used with Kraken). The *k*-mer count thresholds worked very well, particularly for the biological datasets (Table 1 and Additional file 2: Table S3). On the genus level, the average recall in the biological datasets increases by 4–9%, and the average F1 score increases 2–3%. On the species level, the average increase in recall in the biological sets is between 3 and 12%, and the F1 score increases by 1–2%.

**Table 1:** Performance of read count and unique *k*-mer thresholds at genus and species rank on 10 biological and 21 simulated datasets against the three databases ‘orig’, ‘std’ and ‘nt’

Data Type	Rank	Statistic	orig			std			nt		
			reads	*k*-mers	%diff	reads	*k*-mers	%diff	reads	*k*-mers	%diff
Bio	Genus	Recall	0.90	**0.93**	+4.0%	0.89	**0.94**	+6.2%	0.91	**0.99**	+8.9%
		F1	0.95	**0.96**	+1.8%	0.95	**0.97**	+2.6%	0.96	**0.99**	+3.4%
	Species	Recall	0.85	**0.87**	+2.6%	0.70	**0.78**	+11.8%	0.95	**0.98**	+3.1%
		F1	0.94	0.94	+0.7%	0.90	**0.92**	+2.5%	0.97	**0.99**	+1.6%
Sim	Genus	Recall	**0.96**	0.94	-2.1%	0.95	**0.97**	+2.5%	0.98	0.99	+0.8%
		F1	0.98	0.98	-0.0%	0.98	0.98	+0.3%	0.99	0.99	+0.3%
	Species	Recall	0.92	0.93	+0.6%	0.88	0.88	+0.3%	0.90	0.90	-0.1%
		F1	0.97	0.97	+0.3%	0.94	0.94	+0.5%	0.96	0.96	-0.1%

Bold values indicate better performance by at least 1% difference in the test statistic, show in the third column %diff. Unique *k*-mer count thresholds give up to 10% better recall and F1 scores, particularly for the biological datasets

On the simulated datasets, the differences are less pronounced and vary between databases, even though on average the unique *k*-mer count is again better. However, only in two cases (genus recall on databases “orig” and “std”) the difference is higher than 1% in any direction. We find that simulated datasets often lack false positives with a decent number of reads but a lower number of unique *k*-mer counts, which we see in real data. Instead, in most simulated datasets, the number of unique *k*-mers is linearly increasing with the number of unique reads in both true and false positives (Additional file 1: Figure S3). In biological datasets, sequence contamination and lower read counts for the true positives make the task of separating true and false positives harder.

#### Comparison of KrakenUniq with 11 other methods

Next, we compared KrakenUniq’s unique *k*-mer counts with the results of 11 metagenomics classifiers from McIntyre et al. [15], which include the alignment-based methods Blast + Megan [16, 17], Diamond + Megan [17, 18], and MetaFlow [19]; the *k*-mer-based CLARK [20], CLARK-S [21], Kraken [9], LMAT [22], and NBC [23]; and the marker-based methods GOTTCHA [24], MetaPhlAn2 [25], and PhyloSift [26]. KrakenUniq with database “nt” has the highest average recall and F1 score across the biological datasets, as shown in Table 2. As seen before, using unique *k*-mer instead of read counts as thresholds increases the scores. While the database selection proves to be very important (KrakenUniq with database “std” is performing 10% worse than KrakenUniq with database “nt”), only Blast has higher average scores than KrakenUniq with *k*-mer count thresholds on the original database. On the simulated datasets, KrakenUniq with the “nt” database still ranks at the top, though, as seen previously, there is more variation (Additional file 1: Table S4). Notably, CLARK is as good as KrakenUniq, but Blast has much worse scores on the simulated datasets.

**Table 2:** Performance of KrakenUniq (with unique *k*-mer count thresholds) compared to metagenomics classifiers [15] on the biological datasets (*n* = 10). F1 and recall show the average values over the datasets. Note that “KrakenUniq reads” would be equivalent to standard Kraken

	Genus		Species		
	F1	Recall	F1	Recall	Avg
KrakenUniq nt *k*-mers	**0.99**	**0.99**	**0.99**	**0.98**	**0.99**
KrakenUniq nt reads	0.96	0.91	0.97	0.95	0.95
BlastMeganFilteredLiberal	0.97	0.94	0.97	0.89	0.94
BlastMeganFiltered	0.97	0.93	0.96	0.87	0.93
KrakenUniq orig *k*-mers	0.96	0.93	0.94	0.87	0.93
ClarkM4Spaced	0.95	0.90	0.94	0.88	0.92
KrakenUniq orig reads	0.95	0.90	0.94	0.85	0.91
Kraken	0.95	0.90	0.94	0.84	0.91
KrakenUniq std. *k*-mers	0.97	0.94	0.92	0.78	0.90
DiamondMegan_sensitive	0.98	0.93	0.92	0.74	0.89
KrakenFiltered	0.95	0.91	0.90	0.75	0.88
ClarkM1Default	0.94	0.85	0.91	0.77	0.87
KrakenUniq std. reads	0.95	0.89	0.90	0.70	0.86
LMAT	0.97	0.93	0.91	0.60	0.85
DiamondMegan	0.94	0.87	0.91	0.66	0.85
Gottcha	0.91	0.84	0.87	0.67	0.82
NBC	0.87	0.76	0.85	0.73	0.80
Metaphlan	0.94	0.89	0.83	0.55	0.80
MetaFlow	0.66	0.53	0.65	0.51	0.59
PhyloSift	0.68	0.29	0.78	0.54	0.57
PhyloSift90pct	0.68	0.30	0.77	0.52	0.57

Bold values indicate the highest value in each column

### Generating a better test dataset and selecting an appropriate *k*-mer threshold

In the previous section, we demonstrated that KrakenUniq gives better recall and F1 scores than other classifiers on the test datasets, given the correct thresholds. How can the correct thresholds be determined on real data with varying sequencing depths and complex communities? The test datasets are not ideal for that the biological datasets lack complexity with a maximum of 25 species in some of the samples, while the simulated samples lack the features of biological datasets.

We thus generated a third type of test dataset by sampling reads from real bacterial isolate sequencing runs, of which there are tens of thousands in the Sequence Read Archive (SRA). That way, we created a complex test dataset for which we know the ground truth, with all the features of real sequencing experiments, including lab contaminants and sequencing errors. We selected 280 SRA datasets from 280 different bacterial species that are linked to complete RefSeq genomes (see Additional file 1: Suppl. Methods Section 2.4). We randomly sampled between 1 hundred and 1 million reads (logarithmically distributed) from each experiment, which gave 34 million read pairs in total. Furthermore, we sub-sampled 5 read sets with between 1 and 20 million reads. All read sets were classified with KrakenUniq using the ‘“std” database.

**Fig. 3:**
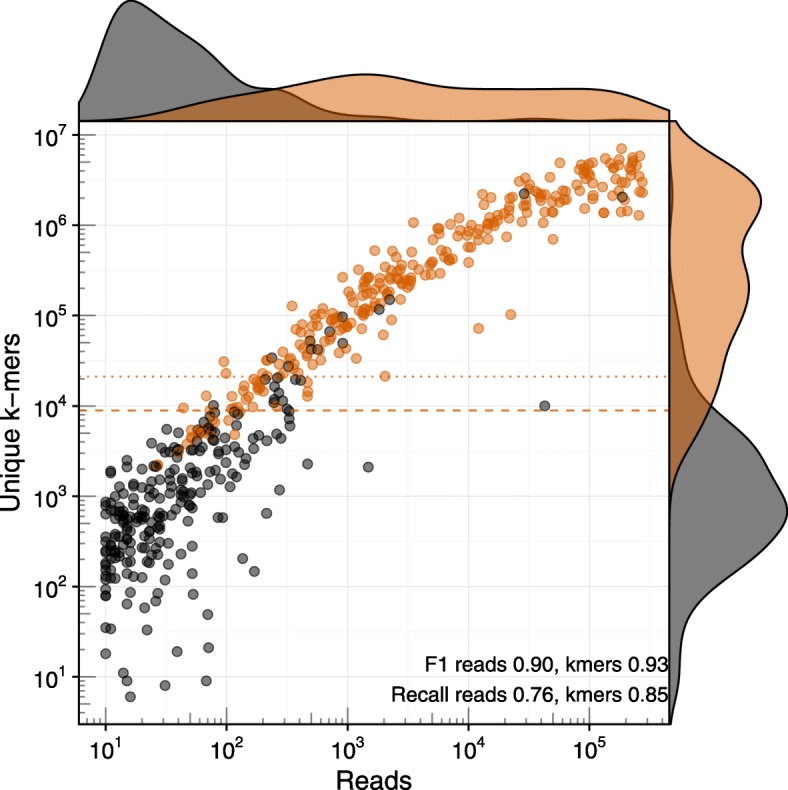
Unique *k*-mer count separates true and false positives better than read counts in a complex dataset with ten million reads sampled from SRA experiments. Each dot represents a species, with true species in orange and false species in black. The dashed and dotted lines show the *k*-mer thresholds for the ideal F1 score and recall at a maximum of 5% FDR, respectively. In this dataset, a unique *k*-mer count in the range 10,000–20,000 would give the best threshold for selecting true species

**Fig. 4:**
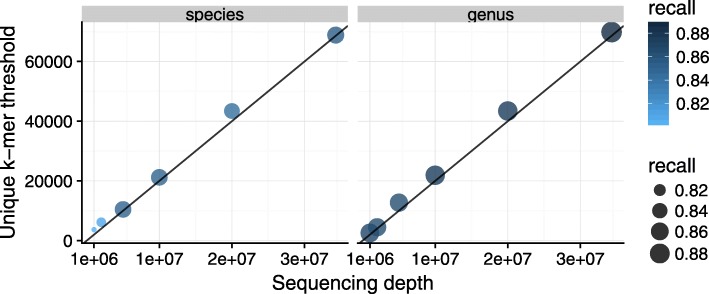
Deeper sequencing depths require higher unique *k*-mer count thresholds to control the false-positive rate and achieve the best recall. A minimum threshold of about 2000 unique *k*-mer per a million reads gives the best results in this dataset (solid line in plot), see Additional file 3: Table S8 for more details

Consistent with the results of the previous section, we found that unique *k*-mer counts provide better thresholds than read counts both in terms of F1 score and recall in all test datasets (e.g., Fig. 3 on 10 million reads—species recall using *k*-mers is 0.85, recall using reads 0.76). With higher sequencing depth, the recall increased slightly—from 0.80 to 0.85 on the species level and from 0.87 to 0.89 on the genus level. The ideal values of the unique *k*-mer count thresholds, however, vary widely with different sequencing depths. We found that the ideal thresholds increase by about 2000 unique *k*-mers per 1 million reads (see Fig. 4). McIntyre et al. [15] found that *k*-mer-based methods show a positive relationship between sequencing depths and misclassified reads. Our analysis also shows that with deeper sequencing depths, higher thresholds are required to control the false-positive rate.

In general, we find that for correctly identified species, we obtain up to approximately *L*-*k* unique *k*-mers per each read, where *L* is the read length because each read samples a different location in the genome. (Note that once the genome is completely covered, no more unique *k*-mers can be detected.) Thus, the *k*-mer threshold should always be several times higher than the read count threshold. For the discovery of pathogens in human patients, discussed in the next section, a read count threshold of 10 and unique *k*-mer count threshold of 1000 eliminated many background identifications while preserving all true positives, which were discovered from as few as 15 reads.

### Exact counting versus estimated cardinality

KrakenUniq’s unique *k*-mer count is an estimate, raising the following question: does using an estimate—instead of the exact count—affect the classification performance?

To answer this question, we implemented an exact counting mode in KrakenUniq. As expected, exact counting requires significantly more memory and runtime. On the full test dataset (with 34.3 mio paired reads sampled from 280 WGS experiments on bacterial isolates), the more efficient of two version of exact counting required 60% more memory and over 200% more runtime. At the same time, we observed virtually no improvement in term of classification performance (Table 3). A likely explanation for this finding is that over- or underestimation of the true cardinality by a small amount (e.g., 1%) rarely changes the ranking of the identifications. There will be cases, however, where a true species may fall just under a threshold due to the estimation error, and users may choose to use exact counting with KrakenUniq, although this will incur a large penalty in both runtime and memory consumption.

**Table 3:** Using cardinality estimates does not decrease classification performance on the test dataset. KrakenUniq in the default mode—using HyperLogLog cardinality estimation with precision 14—classifies reads as accurately as KrakenUniq using exact counting, on both the species and genus level. (Only genus level is shown in the table, which also shows Kraken’s performance for comparison). Note that we tested two versions of exact counting. In version 1, we implemented exact counting using C++ standard library’s unordered_set. Most time is spent on merging counters in the end for report generation. In version 2, we implemented exact counting using khash from klib (https://github.com/attractivechaos/klib/). KrakenUniq uses version 2. Both unordered sets and the hash map require heap allocations for updating, which can cause significant performance cost at runtime because of global locks. Wall clock time for KrakenUniq includes report generation (which takes an additional 2m33s for Kraken)

	Kraken	KrakenUniq		
		Default	Exact(1)	Exact(2)
Computational performance				
Wall clock time^3^	17m38s	**14m18s**	3h30m6s	45m30s
Speed [Mbp/m]	478.4	**595.4**	95.9	377.8
Memory [GB]	**167.1**	168.2	466.2	272.4
Minor page faults × 10^6^	203.5	**192.2**	272.5	904.6
Classification performance				
Recall	0.827	**0.888**	**0.888**	**0.888**
F1 score	0.922	**0.935**	**0.935**	**0.935**

Bold values indicate the highest or lowest values in each row

### Results on biological samples for infectious disease diagnosis

Metagenomics is increasingly used to find species of low abundance. A special case is the emerging use of metagenomics for the diagnosis of infectious diseases [27, 28]. In this application, infected human tissues are sequenced directly to find the likely disease organism. Usually, the vast majority of the reads match (typically 95–99%) the host, and sometimes fewer than 100 reads out of many millions of reads are matched to the target species. Common skin bacteria from the patient or lab personnel and other contamination from sample collection or preparation can easily generate a similar number of reads, and thus mask the signal from the pathogen.

To assess if the unique *k*-mer count metric in KrakenUniq could be used to rank and identify pathogen from human samples, we reanalyzed ten patient samples from a previously described series of neurological infections [4]. That study sequenced spinal cord mass and brain biopsies from ten hospitalized patients for whom routine tests for pathogens were inconclusive. In four of the ten cases, a likely diagnosis could be made with the help of metagenomics. To confirm the metagenomics classifications, the authors in the original study re-aligned all pathogen reads to individual genomes.

Table 4 shows the results of our reanalysis of the confirmed pathogens in the four patients, including the number of reads and unique *k*-mers from the pathogen, as well as the number of bases covered by re-alignment to the genomes. Even though the read numbers are very low in two cases, the number of unique *k*-mers suggests that each read matches a different location in the genome. For example, in PT8, 15 reads contain 1570 unique *k*-mers, and re-alignment shows 2201 covered base pairs. In contrast, Table 5 shows examples of identifications from the same datasets that are not well-supported by *k*-mer counts. We also examined the likely source of the false-positive identifications by blasting the reads against the full nt database, and found rRNA of environmental bacteria, human RNA, and PhiX-174 mis-assignments (see Additional file 1: Suppl. Methods for details). Notably, the common laboratory and skin contaminants PhiX-174, *Escherichia coli*, *Cutibacterium acnes*, and *Delftia* were detected in most of the samples, too (see Additional file 1: Table S6). However, those identifications are solid in terms of their *k*-mer counts—the bacteria and PhiX-174 are present in the sample, and the reads cover their genomes rather randomly. To discount them, comparisons against a negative control or between multiple samples are required (e.g., with Pavian [29]).

**Table 4:** Validated pathogen identifications in patients with neurological infections have high numbers of unique *k*-mers per read. The pathogens were identified with as few as 15 reads, but the high number of unique *k*-mers indicates distinct locations of the reads along their genomes. Re-alignment of mapped reads to their reference genomes (column “Covered bases”) corroborates the finding of the unique *k*-mers (see also Additional file 1: Figure S4). Interestingly, the *k*-mer count in PT5 indicates that there might be multiple strains present in the sample since the *k*-mers cover more than one genome. Read lengths were 150–250 bp

Sample	Matched microorganism	Reads	*k*-mers	Covered bases
PT5	Human polyomavirus 2	9650	7129	5130/5130
PT7	*Elizabethkingia genomo* sp. 3	403	20,724	53,256/4,433,522
PT8	*Mycobacterium tuberculosis*	15	1570	2227/4,411,532
PT10	Human gammaherpesvirus 4	20	2084	2822/172,764

**Table 5:** False-positive identifications have few unique *k*-mers. Using an extended taxonomy, the identifications in PT4 and PT10 were matched to single accessions (instead of to the species level). The likely true source of the mapped sequences was determined by subsequent BLAST searches and included 16S rRNA present in many uncultured bacteria, human small nucleolar RNAs (snRNAs), and phiX174

Sample	Matched microorganism	Reads	*k*-mers	Source
PT3	*Clostridioides difficile*	122	126	16S rRNA
PT4	Hepatitis C virus JF343788.1 recombinant hepatitis C virus	101	3	Human snRNA
PT5	*Akkermansia muciniphila*	936	136	16S rRNA
PT10	Human betaherpesvirus 5 JN379815.1 human herpesvirus 5 strain U04, partial genome	63	5	phiX174

#### Further extensions in KrakenUniq

KrakenUniq adds three further notable features to the classification engine.

1. Enabling strain identification by extending the taxonomy: The finest level of granularity for Kraken classifications are nodes in the NCBI taxonomy. This means that many strains cannot be resolved, because up to hundreds of strains share the same taxonomy ID. KrakenUniq allows extending the taxonomy with virtual nodes for genomes, chromosomes, and plasmids, and thus enabling identifications at the most specific levels (see Additional file 1: Suppl. Methods Section 3)
2. Integrating 100,000 viral strain sequences: RefSeq includes only one reference genome for most viral species, which means that a lot of the variation of viral strain is not covered in a standard RefSeq database. KrakenUniq sources viral strain sequences from the NCBI Viral Genome Resource that are validated as “neighbors” of RefSeq viruses, which leads to up to 20% more read classifications (see Additional file 1: Suppl. Methods Section 4).
3. Hierarchical classification with multiple databases: Researchers may want to include additional sequence sets, such as draft genomes, in some searches. KrakenUniq allows to chain databases and match each *k*-mer hierarchically, stopping when it found a match. For example, to mitigate the problem of host contamination in draft genomes, a search may use the host genome as the first database, then complete microbial genomes then draft microbial genomes. More details are available in Additional file 1: Suppl. Method Section 5.

### Timing and memory requirements

The additional features of KrakenUniq come without a runtime penalty and very limited additional memory requirements. In fact, due to code improvements, KrakenUniq often runs faster than Kraken, particularly when most of the reads come from one species. On the test datasets, the mean classification speed in million base pairs per minute increased slightly from 410 to 421 Mbp/m (see Additional file 2: Table S3). When factoring in the time needed to summarize classification results by Kraken-report, which is required for Kraken but part of the classification binary of KrakenUniq, KrakenUniq is on average 50% faster. The memory requirements increase on average by 0.5 GB from 39.5 to 40 GB.

On the pathogen ID patient data, where in most cases over 99% of the reads were either assigned to human or synthetic reads, KrakenUniq was significantly faster than Kraken (Additional file 1: Table S5). The classification speed increased from 467 to 733 Mbp/m. The average wall time was about 44% lower, and the average additional memory requirements were less than 1 GB, going from 118.0 to 118.4 GB. All timing comparisons were made after preloading the database and running with 10 parallel threads.

## Discussion

In our comparison, KrakenUniq performed better in classifying metagenomics data than many existing methods, including the alignment-based methods Blast [16], Diamond [30], and MetaFlow [19]. Blast and Diamond results were post-processed by Megan [17, 31], which assigns reads to the lowest common ancestor (LCA), but ignores coverages when computing the resulting taxonomic profile. Thus, the taxonomic profile (with read counts as abundance measures) is sensitive to over-representing false positives that have coverage spikes in parts of the genome in the same way as non-alignment based methods. Coverage spikes may appear due to wrongly matched common sequences (e.g., 16S rRNA), short amplified sequences floating in the laboratory, and contamination in database sequences. MetaFlow, on the other hand, implements coverage-sensitive mapping, which should give better abundance calls, but it did not perform very well in our tests. Going from alignments to a good taxonomic profile is difficult because coverage information cannot be as easily computed for the LCA taxon and summarized for higher levels in the taxonomic tree. In comparison, reads and unique *k*-mer counts can be assigned to the LCA taxa and summed to higher levels. Notably, KrakenUniq’s *k*-mer counting is affected by GC biases in the sequencing data the same way as other read classifiers and aligners [32] and may underreport GC-rich or GC-poor genomes.

## Conclusions

KrakenUniq is a novel method that combines fast *k*-mer-based classification with an efficient algorithm for counting the number of unique *k*-mers found in each species in a metagenomics dataset. When the reads from a species yield many unique *k*-mers, one can be more confident that the taxon is truly present, while a low number of unique *k*-mers suggests a possible false-positive identification. We demonstrated that using unique *k*-mer counts provides improved accuracy for species identification and that *k*-mer counts can help greatly in identifying false positives. In our comparisons with multiple other metagenomics classifiers on multiple metagenomics datasets, we found that KrakenUniq consistently ranked at the top. The strategy of counting unique *k*-mer matches allows KrakenUniq to detect that reads are spread across a genome, without the need to align the reads. By using a probabilistic counting algorithm, KrakenUniq is able to match the exceptionally fast classification time of the original Kraken program with only a very small increase in memory. The result is that KrakenUniq gains many of the advantages of alignment at a far lower computational cost.

## Additional files

Additional file 1: Supplementary materials. Contains supplementary methods sections 1–7, **Figures S1–S4**, and **Tables S1–S2, S4–S7**. (PDF 1105 kb)

Additional file 2: **Table S3**, which has the description and results of the test datasets from McIntyre et al. (2017). (XLSX 18 kb)

Additional file 3: **Table S8**, showing the comparison of sequencing depth and *k*-mer count threshold from Fig. 4. (PDF 93 kb)
