# Supplementary material for: KrakenUniq: confident and fast metagenomics classification using unique k-mer counts
Source: Genome Biol. 2018 Nov 16;19:198. doi: 10.1186/s13059-018-1568-0 (PMC6238331; doi:10.1186/s13059-018-1568-0)
Supplement: Supplementary file 1 — Supplementary materials. Contains supplementary methods sections 1–7, Figures S1–S4, and Tables S1–S2, S4–S7. (PDF 1105 kb) [file 13059_2018_1568_MOESM1_ESM.pdf]

SUPPLEMENTARY INFORMATION FOR KRAKENUNIQ
BREITWIESER, BAKER AND SALZBERG, 2018

1. HyperLogLog algorithm - from Flajolet to Heule to Ertl 2

2. Dataset analysis 8

2.1 Building of std and nt databases 8

2.2 Kraken and KrakenUniq command lines 10

2.3 McIntyre et al., 2017 [8] dataset analysis........ 11

2.4 Synthetic community sampled from SRA isolate experiments 16

2.5 Salzberg et al., 2016 [9] dataset analysis 17

3. Storing strain genomes with assembly project and sequence accessions 20

4. Integrating viral strain genomes in the database 22

5. Hierarchical read classification with multiple databases 23

6. Switching from Kraken to KrakenUniq 23

7. New taxonomy database format........ 24

8. References for the Supplement 25

1. HyperLogLog algorithm - from Flajolet to Heule to Ertl

HyperLogLog is a probabilistic unique count (cardinality) estimator of streams of values with duplicates. It stores a sketch of the data in a concise structure and is very accurate for small cardinalities, keeps constant accuracy rates for up to very high cardinalities.

Intuition. A random bit-string of length n can be seen as the outcome of n independent binomial trials with $p = 0.5$. Let k be the position of the first 1-bit, i.e. the bit string starts with (k-1) 0-bits before the first 1-bit. Since the bits are independent, the probability of k is the product of the probabilities, 0.5^k. For example, $k = 6$ means that the bit string starts with 00001_2, and the probability of a random bit-string conforming to the pattern is 0.5^6 or 1/64 (**Table S1**).

k	Pattern_k	P_k	E_k
1	1xxxxxxxxxxx..x	0.5	2
2	01xxxxxxxxxx..x	0.25	4
3	001xxxxxxxxx..x	0.125	8
4	0001xxxxxxxx..x	0.0625	16
l	$0^{l-1}1x^{n-l}$	2^{-l}	2^l

Table S1: The probabilities observing the first 1-bit at position k in a random bit string. E_k shows the expected number of bit-strings we have to observe until seeing one with Pattern_k, and is $1/P_k$.

Inversely, the expected number of independent bit-strings until we see k, E_k is 2^k. If we knew only the maximum number of k, $k_{\max}$, in a stream of independent random bit strings, the best guess at the cardinality of the stream is $2^{k\max}$. Note that this statistic discounts duplicates, as duplicates have the same value. To achieve high precision, HyperLogLog first distributes the stream hashes into $2^p=m$ registers based on the first p bits. The latter 64-p bits are used to determine $k_{\max}$ of that register (assuming 64-bit

hashes). The final estimate is calculated as harmonic mean of the estimates of all registers. The relative error of the estimate is about $2^{-p/2}$ (see **Fig. 2** in the main text).

Algorithm and Implementation. Using 2-bits per base, k-mers up to 31 base pairs can be stored in 64 bits. As k-mers are neither random, nor independently distributed, we hash the k-mers to distribute them uniformly. Good hash functions (a) distribute the input evenly across the output range, and (b) create very different outputs for close inputs (avalanche effect). If both properties are fulfilled for the input (k-mers from different genomes), then we can expect to see precise estimates.

KrakenUniq implements a version of HyperLogLog with the following modifications:

- 64-bit hashes are created by the fast finalizer of the MurMurHash3 algorithm (Appleby, 2017)
- For smaller cardinalities (up to 2^{p-2}) we use a sparse representation that encodes hashes with a much higher precision [1]
- The final estimate is calculated form the register values based on an improved formula [2]
- The counters can be easily merged for parallel execution. KrakenUniq gives sets of reads to workers, which return HLL sketches in addition to the classification results. The sketches of each taxon are merged into their master sketches by taking the maximum of all register values

Computing the estimate. KrakenUniq implements the recently derived improved estimator for HyperLogLog sketches [2]. Previously proposed methods, including [3] and [1], require empirically determined thresholds to account for biases and switching between linear counting and HLL estimator. However, as [2] shows, the empiric bias correction does not always work.

The raw estimate $\hat{e}_{\text{raw}}$ of Flajolet is based on the harmonic mean of the estimates of the individual registers, times a bias correction factor α_m. Following the notation of [2], $C:=(C_0, \ldots, C_{q+1})$ is the register histogram, where C_k is the number of registers in M that have the value k.

$$\hat{e}_{\text{raw}} = \frac{\alpha_m m^2}{\sum_{k=0}^{q+1} C_k 2^{-k}}$$

While this works well when the true cardinality λ is in the range $2^p << \lambda << 2^{p+q}$, the estimator is severely biased outside of this range. To account for small range errors, the Flajolet estimator uses linear counting [4] below a threshold of $2.5 \cdot 2^p$:

$$\hat{e}_{\text{small}} = m \log m / C_0$$

While the linear counting estimate is very accurate up to that threshold, the raw estimate that is used above the threshold is still very biased. This can be seen in a big spike in errors in the Flajolet estimate (**Fig. S1**). Heule et al. propose empirically determined bias tables to get rid of the bias. Using observed biases in big amount of random data, they provide correction factors along 200 interpolation points when the raw estimator is in range $\sim 2^p < \hat{e}_{\text{raw}} < 5 \cdot 2^p$. Mostly this correction manages to get rid of the bias (**Fig. S2**), however in some ranges the bias persists (**Fig. S2**).

For large range errors, Flajolet proposes a correction factor when hitting raw estimates above $1/30\, 2^{32}$ (with 32-bit hashes). That factor, however, does not solve the problem but just flips the bias in the opposite direction [2]. When using 64-bit hashes and counting way below 2^{64}, though this bias can be largely ignored [1].

[2] describes how the biases occur due to not accounting for the fact that the register values are censored at 0 and q+1. Based on the expectation of the censored registers C_0 and C_{q+1}, Ertl derives an improved formula for the estimator without bias:

$$\hat{e}_{\text{ertl}} = \frac{\alpha_\infty m^2}{\sum_{k=1}^{q} C_k 2^{-k} + m\sigma(C_0\, m) + m2^{-q}\tau(1 - C_{q+1}/m)},$$

with

$$\sigma(x) := x + \sum_{k=1}^{\infty} x^{2^k} 2^{k-1},$$

$$\tau(x) := 1 - x - \sum_{k=1}^{\infty} \left(1 - x^{2^{-k}}\right) 2^{-k},$$

$$\alpha_{\infty} := 1/2 \log 2$$

As seen in **Figures S1 and S2**, the improved estimator of Ertl does not demonstrate any bias. Furthermore, using the sparse representation of Heule et al. for smaller cardinalities gives great precision for lower cardinalities.

Performance testing: The HyperLogLog implementation was tested both on random numbers (**Figures S1 and S2**) and actual database k-mers (**Figure 2** in the main text). The plots were created with R [5] and ggplot2 [6]. All code for recreating the estimates and plots is available at https://github.com/fbreitwieser/krakenuniq-manuscript-code.

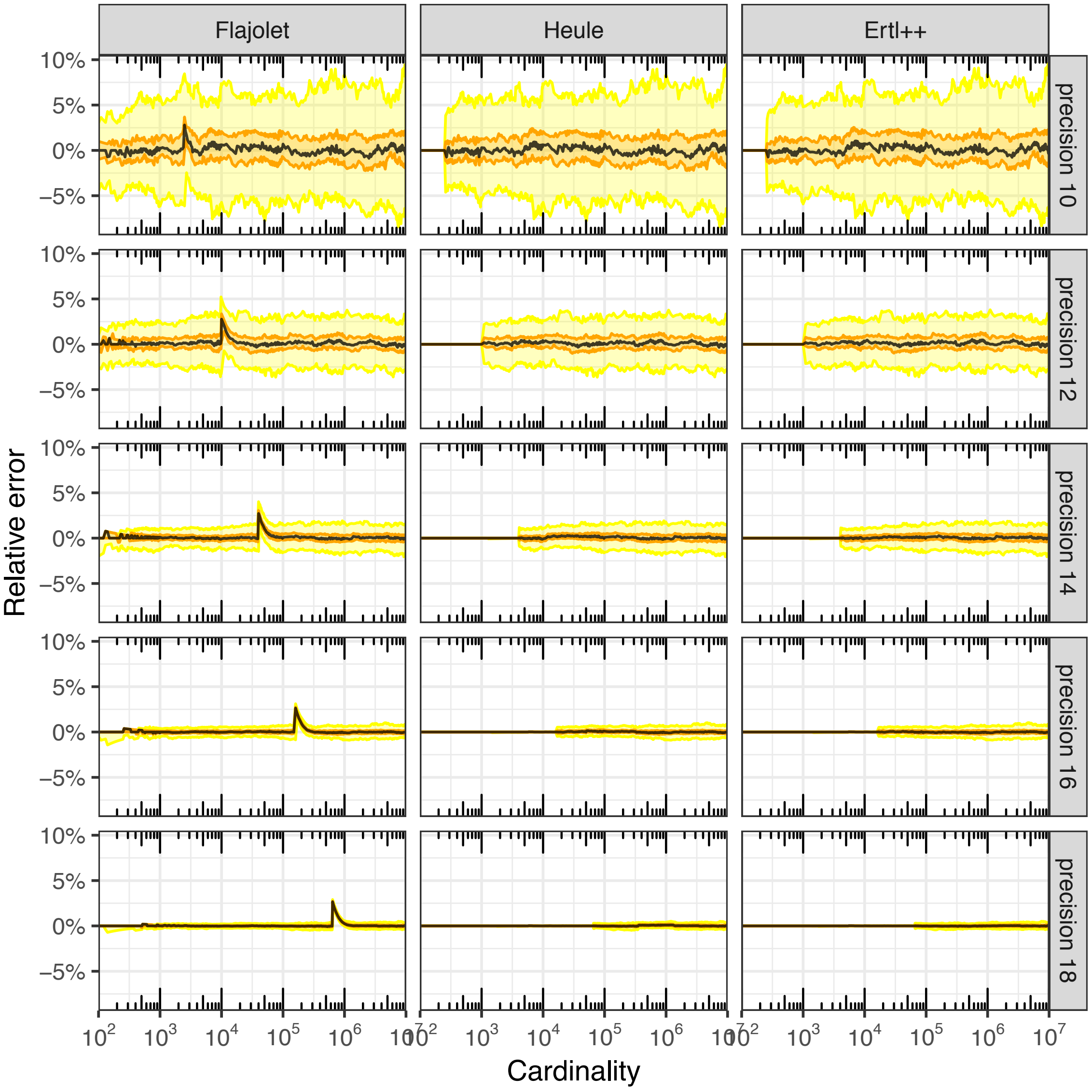

Figure S1: Comparison of relative errors with Flajolet, Heule and Ertl estimators with varying values of *p*. Black line: median relative error, orange lines 68.2% percentiles, yellow lines 95% percentiles. As expected, the relative error goes down with higher precision values. For both Heule's and Ertl's estimator we use sparse representation for cardinalities up to $2^{p\text{-}2}$ (p'= 25). Note that the empirical bias correction of Heule and the mathematical correction of Ertl both get rid of the big spike apparent for Flajolet, when the estimator switches between linear counting and HLL counting. Data from 100 simulated random number runs (64-bit Mersenne Twister seeded with system entropy).

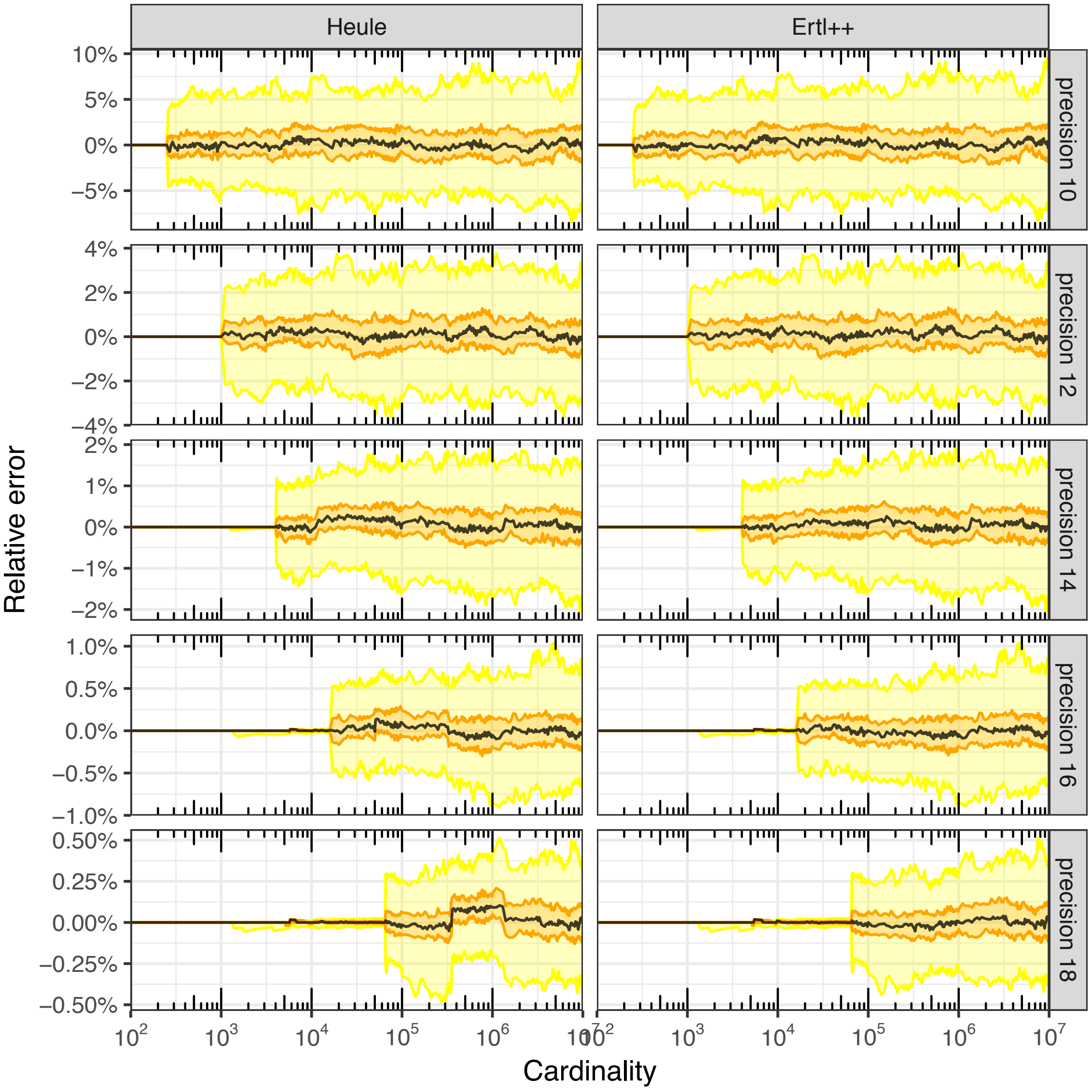

Figure S2: Comparison of Heule and Ertl estimators with sparse representation and variable y-axis. At certain precisions and cardinalities, the empirical bias correction values of Heule are not working well. For precision 16, bias is present around cardinalities of 15,000, and for precision 18, bias is present around cardinalities of 150,000 to 1,000,000. Legend: Black line is median relative error, orange lines encompass 68.2% of the estimate errors, yellow lines encompass 95% of the estimate errors. Data from 100 randomly simulated runs of numbers.

2. Dataset analysis

2.1 Building of std and nt databases

KrakenUniq includes the new krakenuniq-download script to download and dust genomes from specific domains from RefSeq and Genbank. For example, the following command downloads the genomic and RNA sequences for all chromosome-level assembled genomes in the category ‘vertebrate_mammalian’ with taxID 9606 - which gives the two human genomes GRCh38.p11 and CHM1_1.1.1:

```
krakenuniq-download --db DB_DIR --fna rna,genomic
refseq/vertebrate_mammalian/Chromosome/taxid9606
```

For the reanalysis of the data, we made two databases, ‘std’ and ‘nt’, both with a *k* of 31.

(A) ‘std’ (downloaded and built October 26, 2017): Includes artificial sequences from UniVec and EmVec, all complete viral, archaeal and bacterial genomes from RefSeq, the two human genomes mentioned above, and viral strain sequences. All microbial sequences were dusted with NCBI dustmasker. Command line:

```
krakenuniq-download --db DB_DIR taxonomy contaminants

krakenuniq-download --db DB_DIR --dust --include-viral-neighbors refseq/viral/Any

krakenuniq-download --db DB_DIR --dust refseq/archaea refseq/bacteria

krakenuniq-download --db DB_DIR --fna rna,genomic
refseq/vertebrate_mammalian/Chromosome/taxid9606



krakenuniq-build --db DB_DIR --build --taxids-for-genomes --taxids-for-sequences --threads
10
```

The database contains 8113 genomes from 3048 prokaryotic species and 139477 sequences from 7295 viral or viroid species. The database construction took 18 hours with 10 threads and uses 169 GB of disk

space + 8.1GB for the index. The samples were run on a machine with four Intel Xeon CPUs E7- 4830 (eight cores each) and 1TB of RAM. Number of sequences and unique k-mers in this database:

Name	Rank	TaxID	# of sequences	# of *k*-mers	*k*-mer duplication
Bacteria	superkingdom	2	15382	1.35E+10	2.27
Homo sapiens	species	9606	232355	2.67E+09	2.49
Archaea	superkingdom	2157	369	4.75E+08	1.26
Viruses	superkingdom	10239	139477	2.94E+08	4.47
synthetic construct	species	32630	9740	2.39E+06	3.91

(B) 'nt' is based on the microbial part of nt, downloaded from ftp://ftp.ncbi.nih.gov/blast/db/FASTA/nt.gz on February 23, 2018. The nt sequences were filtered based on the taxon list provided by Kaiju [7] at https://github.com/bioinformatics-centre/kaiju/blob/master/util/taxonlist.tsv. The table below shows the included taxa and the number of sequences and distinct k-mers in their clade:

Name	Rank	TaxID	# of sequences	# of *k*-mers	*k*-mer duplication
Bacteria	superkingdom	2	6549136	1.72E+10	2.67
Fungi	kingdom	4751	3836040	4.85E+09	1.76
Alveolata	no rank	33630	472264	8.47E+08	1.68
Apicomplexa	phylum	5794	299510	6.97E+08	1.77
Archaea	superkingdom	2157	326186	6.64E+08	1.38
Viruses	superkingdom	10239	2050013	5.42E+08	6.49
Euglenozoa	no rank	33682	150499	3.19E+08	2.09
Kinetoplastida	order	5653	146387	3.14E+08	2.1
Stramenopiles	no rank	33634	330637	3.12E+08	1.47
Chlorophyta	phylum	3041	159680	2.35E+08	1.2
Amoebozoa	no rank	554915	133625	1.45E+08	1.23
Rhodophyta	no rank	2763	60582	6.93E+07	1.52
Choanoflagellida	order	28009	21662	4.46E+07	1.08
Parabasalia	no rank	5719	57906	3.74E+07	1.47
Entamoeba	genus	5758	41528	3.45E+07	1.44
Cryptophyta	class	3027	29269	3.40E+07	1.09

Haptophyceae	no rank	2830	41463	3.08E+07	1.44
Heterolobosea	class	5752	16885	2.40E+07	1.07
Apusozoa	no rank	554296	10877	2.07E+07	1.06
Fornicata	no rank	207245	14657	9.35E+06	1.45
Rhizaria	no rank	543769	19683	8.83E+06	2.07
Jakobida	no rank	556282	459	1.39E+06	1.09
Glaucocystophyceae	class	38254	352	6.69E+05	1.16
Syndiniales	order	88547	2317	3.39E+05	2.12
Oxymonadida	order	66288	472	2.35E+05	2.24
Malawimonadidae	family	136087	43	1.58E+05	1.04
Centroheliozoa	no rank	193537	295	1.34E+05	1.59
Telonemida	order	589438	177	8.36E+04	1.21
Palpitomonas	genus	759891	14	8.20E+04	1.06
Collodictyonidae	family	190322	22	7.55E+04	1.52
Picozoa	phylum	419944	104	6.48E+04	1.21
Tsukubamonadidae	family	1084709	9	5.54E+04	1
Katablepharidophyta	class	339960	248	4.57E+04	2.36
Breviatea	no rank	1401294	21	1.92E+04	1.03
Trimastix	genus	137418	13	1.29E+04	1.03

Command line:

```
make -f KRAKENHLL_DIR/microbial-nt-db/Makefile DODUST=0 nt
```

The resulting database is 234GB in size plus 8.1 GB for the index.

2.2 Kraken and KrakenUniq command lines

For all comparisons of Kraken and KrakenUniq, the databases were preloaded with kraken --preload.

The following command line was used for KrakenUniq:

```
krakenuniq --db DB_DIR --threads 10 --report-file SAMPLE.krakenuniq.report.tsv --fastq --
gzip SAMPLE.fq.gz > SAMPLE.krakenuniq.tsv
```

and Kraken v1.0 was run on the same database with:

```
kraken --db DB_DIR --threads 10 --fastq --gzip SAMPLE.fq.gz > SAMPLE.kraken.tsv
```

```
kraken-report --db DB_DIR --threads 10 --report-file SAMPLE.report.tsv --fastq --gzip
SAMPLE.fq.gz > SAMPLE.kraken.tsv
```

Note that the standard output can be turned off by adding --output off to the command line, which leads to a much better runtime.

2.3 McIntyre et al., 2017 [8] dataset analysis

Results for Blast, Clark, Clark Spaced, Diamond, Gottcha, Kraken, LMAT, MetaFlow, MetaPhlAn, NBC and PhyloSift were downloaded from https://pbtech-vc.med.cornell.edu/git/mason-lab/benchmarking_metagenomic_classifier (files species_results_formatted.tar.gz and genus_results_formatted.tar.gz). We reran the samples with KrakenUniq with the two new databases std and nt, as well as the Kraken database 'orig' used in the original comparison of the benchmarking paper. Note that that 'KrakenUniq orig reads' and 'Kraken' results are mostly the same, but there are slight differences as the authors of the benchmarking paper did taxonomy mapping on the results files, too, to achieve a fairer comparison between the methods.

Taxonomy fixes for test datasets

We had fix the truth tables of 13 datasets due to changes in the NCBI taxonomy since the publishing of the datasets. The types of change were merging of species into an existing species, moving of a species to a different genus, moving of subspecies to a different species/genus, and mistaken taxa in the original truth tables. Note that there was no automated way to update the tables - only the subset of merged species is recorded in the NCBI taxonomy dumps. For all other cases, manual investigation was necessary. We validated all changes and made sure that the other methods had the right truth sets, too. Note that new databases are penalized in some cases in other ways. For example, the species *Nanoarchaeum equitans* (LC5) previously had a genome in RefSeq, and was detected in the Kraken analysis of McIntyre et al. But this genome has since been suppressed in RefSeq, and thus is not part of

the ‘std’ database. There are also some discrepancies that we did not fix, as they do not apply to all methods equally. For example, the species *Borreliaella bavariensis* (species taxID 664662, genus taxid 64895) is under its old genus *Borrelia* (genus taxID 138) in the ‘orig’ database, but under *Borreliaella* in the truth sets. While some tools (Blast, Diamond, LMAT, MetaPhlAn) detect *Borrelia* in higher abundance, others (Clark, LMAT, NBC, PhyloSift, original Kraken results) detects correctly *Borreliaella*.

- LC5: *Anabaena variabilis* (taxID 1172) of the dataset Huttenhower LC5 has been merged to *Trichormus variabilis* (species taxID 264691, genus taxID 264688).
- ds.7
    - Both Desulfurococcus fermentans (taxID 228748) and Desulfurococcus kamchatkensis (taxID 477693.7) have been merged into Desulfurococcus amylolyticus, taxID 94694.
    - Chlamydia pneumoniae phage CPAR39, taxID 117575, has been merged to Chlamydia virus CPAR39, taxID 1986029
    - The subspecies *Polynucleobacter necessarius* subsp. asymbioticus (subspecies taxID 576611) was promoted to its own species *Polynucleobacter asymbioticus* (species taxID 576611)
    - The subspecies *Desulfovibrio aespoeensis* Aspo-2 (subspecies taxID 643562, species taxID 182210, genus taxID 872) was moved to the genus *Pseudodesulfovibrio* (same subspecies taxID, same species taxID, genus taxID 2035811)
    - *[Clostridium] sticklandii* (species taxID 1511, genus taxID 1481960) was moved to the genus *Acetoanaerobium* (same species taxID, genus taxID 186831)
- HC1, LC3, UnAmbiguouslyMapped_ds.7: *Spirochaeta smaragdinae* (genus taxID 146) was moved into the genus *Sediminispirochaeta* (genus taxID 1911556, same species taxID). In HC1 and LC3, *S. smaragdinae* is the only species of the genus *Spirochaeta/Sediminispirochaeta*, while ds.7 has other *Spirochaeta* species that stay in their genus.

- UnAmbiguouslyMapped_ds.nycsm: *Enterobacter aerogenes* (species taxID 548) was moved to the genus *Klebsiella* (genus taxID 570, same species taxID). Other Enterobacteria species that stay in their genus are in that dataset, too.
- HMP_even_454, HMP_even_illum, ds.gut, ds.hous2: *Propionibacterium acnes* was moved to the genus *Cutibacterium* (genus taxID 1912216, same species taxID). In ds.7, other Propionibacterium species (that stays in its genus) are present, while in the others *C. acnes* is the only species of the genus *Propionibacterium/Cutibacterium*.
- Carma: *[Haemophilus] parasuis* was moved to the genus *Glaesserella* (genus taxID 2094023, same species taxID 738). This change happened between the times the 'std' and the 'nt' databases were built - thus the changes in this dataset apply only to 'nt'.
- HC1, LC2: *Peptoclostridium difficile* was moved to the genus *Clostridioides* (genus taxID 1870884, same species taxID).
- ds.hous1: The subspecies *Phaeobacter gallaeciensis* 2.10 (taxID 383629) was moved to the species *Phaeobacter inhibens* (species taxID 221822, same subspecies taxID).
- The following changes were made to the general truth tables. These reflect mistakes in the truth tables, and we should get more accurate estimates for all methods using the corrected versions:
    - LC1:
        - *Vibrio harveyi* (species taxID 669) was updated to *Vibrio campbellii* (species taxID 680). All methods identify *V. campbellii* at high levels, and only two (LMAT and PhyloSift) identify *V. harveyi* at very low levels (*V. campbellii* was detected by both methods at 6 and 3000 times higher levels, resp.). The strain of the reads was identified with KrakenUniq as *Vibrio campbellii* ATCC BAA-1116, which was previously known as *Vibrio harveyi* ATCC BAA-1116.
        - *Prosthecochloris vibrioformis* (species taxID 1098, genus taxID 1101) was updated to *Chlorobium phaeovibrioides* (species taxID 1094, genus taxID 1091). The former species was identified only once at very low abundance, while the later was identified by Blast,

CLARK, Diamond, Gottcha, Kraken, LMAT, MetaFlow, MetaPhlAn, NBC and Phylosift at high abundances. The strain of the reads was identified as *Chlorobium phaeovibrioides* DSM 265 by KrakenUniq, which was previously known as *Prosthecochloris vibrioformis* DSM 265.

- RAIphy: Aster yellows phytoplasma (species taxID 35779) was updated to Aster yellows witches'-broom phytoplasma (species taxID 229545, same genus). No method identified the former species, several methods (Blast, Clark, Gottcha, Kraken, LMAT, NBC) identified the later.

We provide patch files for the truth sets in https://github.com/fbreitwieser/krakenuniq-manuscript-code.

[**Table S3** is in a separate file].

Table S4: F1 score and recall of KrakenUniq compared other classifiers on simulated test datasets (n=21).

	Genus		Species		
	F1	Recall	F1	Recall	Avg
KrakenUniq nt kmers	0.99	0.99	0.96	0.90	0.96
KrakenUniq nt reads	0.99	0.98	0.96	0.90	0.96
KrakenUniq orig reads	0.98	0.96	0.97	0.92	0.96
ClarkM1Default	0.98	0.95	0.96	0.92	0.96
KrakenUniq orig kmers	0.98	0.94	0.97	0.93	0.95
KrakenFiltered	0.99	0.93	0.97	0.92	0.95
Kraken	0.99	0.93	0.97	0.92	0.95
KrakenUniq std kmers	0.98	0.97	0.94	0.88	0.95
LMAT	0.99	0.94	0.96	0.87	0.94
KrakenUniq std reads	0.98	0.95	0.94	0.88	0.94
ClarkM4Spaced	0.98	0.93	0.94	0.83	0.92
BlastMeganFilteredLiberal	0.97	0.80	0.96	0.87	0.90
Gottcha	0.93	0.85	0.91	0.82	0.88
BlastMeganFiltered	0.94	0.76	0.92	0.81	0.86
DiamondMegan	0.93	0.77	0.82	0.67	0.80

DiamondMegan_sensitive	0.93	0.77	0.82	0.67	0.80
Metaphlan	0.82	0.63	0.79	0.61	0.71
NBC	0.82	0.62	0.72	0.53	0.67
PhyloSift90pct	0.84	0.56	0.75	0.32	0.62
PhyloSift	0.84	0.54	0.75	0.32	0.61
MetaFlow	0.55	0.44	0.50	0.37	0.46

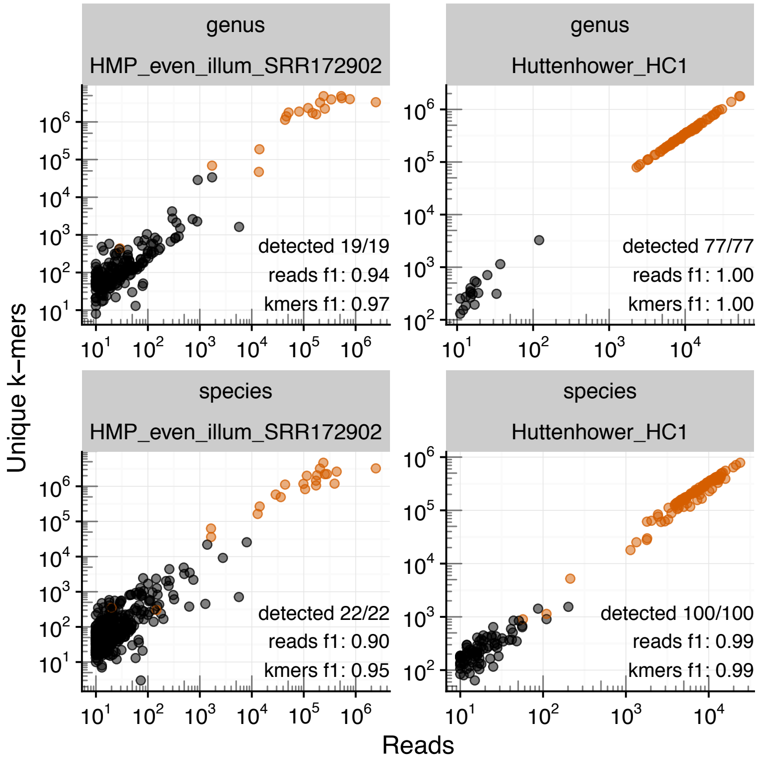

Figure S3: Reads versus k-mers on a biological and a simulated dataset (left and right panes, resp.) on the genus and species level (upper and lower panes, resp.). While the biological dataset does not have many species, there is nearly perfect separation between ‘true’ and ‘false’ taxa in the simulated dataset.

Dataset availability

- Sequence data and ‘truth sets’ for the test datasets is available at https://ftp-private.ncbi.nlm.nih.gov/nist-immsa/IMMSA/

- Results for the classifiers are available at https://pbtech-vc.med.cornell.edu/git/mason-lab/benchmarking_metagenomic_classifiers

2.4 Synthetic community sampled from SRA isolate experiments

We generated a complex artificial bacterial community from actual sequencing reads by sub-sampling reads from isolate sequencing experiments. To get high-quality data, we used isolate sequencing experiments that contributed to completed genomes that are part of RefSeq. We downloaded the assembly summary for all bacterial genomes in RefSeq from ftp://ftp.ncbi.nlm.nih.gov/genomes/refseq/bacteria/assembly_summary.txt and filtered it down to the latest complete genome assemblies that had a BioSample accession (8078 assemblies). Using NCBI eutils, we downloaded the BioSample summary in XML format to get the links to SRA experiments. Using a custom script from http://bioinfo.umassmed.edu/bootstrappers/guides/main/python_get_sra_run_ids.html, we gathered the SRA run information for all 2605 BioSample's with SRA experiment information. For each SRA experiment, we downloaded one SRA run which was Illumina-generated, paired-end and publicly accessible, and classified the reads with KrakenUniq against the 'std' database (in total, we classified 1259 runs). Runs that had at least 75% of the reads assigned to the correct species with more than one million reads were picked for sub-sampling. We sampled between one hundred and one million reads, logarithmically distributed, from each picked run, which totaled 34.3 million paired reads from 259 species. Using seqtk, we randomly sampled about 100 thousand, 200 thousand, 500 thousand, one million, two million and five million reads with mostly the same species composition but less deep sequencing for further analysis.

Dataset availability

The simulated read datasets are available at:

ftp://ftp.ccb.jhu.edu/pub/software/krakenuniq/SraSampledDatasets/

2.5 Salzberg et al., 2016 [9] dataset analysis

Sample	Number of reads	Speed (Mbp/m)			Wall time (m:s)				Max memory (GB)		
		kraken	kraken uniq	speed-up	kraken	kraken-report	kraken uniq	speed-up*	kraken	kraken uniq	increase
PT1	12,022,284	487.89	730.99	49.83%	4:35.75	0:55.40	3:11.18	-42.27%	122.74	123.07	0.27%
PT2	8,294,101	483.66	602	24.47%	8:02.34	1:23.18	5:21.21	-43.20%	113.16	113.50	0.30%
PT3	17,669,644	508.34	698.39	37.39%	5:20.30	0:49.66	4:20.68	-29.54%	132.56	132.93	0.27%
PT4	29,101,779	467.33	812.55	73.87%	8:09.75	1:15.19	5:57.75	-36.67%	117.43	117.78	0.29%
PT5	26,919,065	467.43	798.05	70.73%	8:29.84	1:38.30	4:56.05	-51.32%	119.61	119.97	0.30%
PT6	27,261,739	450.36	776.24	72.36%	8:31.67	1:32.81	5:13.75	-48.10%	119.68	120.04	0.29%
PT7	19,065,574	558.09	819.82	46.90%	8:24.83	1:32.52	5:12.86	-47.63%	116.45	116.82	0.31%
PT8-S1	6,385,699	436.51	725.32	66.16%	3:07.28	0:43.75	2:02.68	-46.90%	104.54	104.87	0.32%
PT8-S2	7,661,802	430.89	726.14	68.52%	2:23.71	0:38.51	1:37.75	-46.36%	109.25	109.58	0.31%
PT9	26,500,914	436.63	722.26	65.42%	2:51.90	0:41.73	2:00.31	-43.68%	124.42	124.78	0.28%
PT10	21,319,274	411.94	656.13	59.28%	9:09.45	1:33.31	5:44.53	-46.40%	118.19	118.53	0.28%

Table S5: Runtime and memory usage for Kraken and KrakenUniq on patient samples [9] running with 10 threads, precision 14 on the std database. * For kraken, kraken-report was run after classification, and the combined time was compared to KrakenUniq (which generates the report while classifying).

Dataset availability

- Data for the patient datasets (without human reads) is available at:

  https://www.ncbi.nlm.nih.gov/bioproject/314149.

A: Human polyomavirus 2 in PT5 (NC_001699.1)

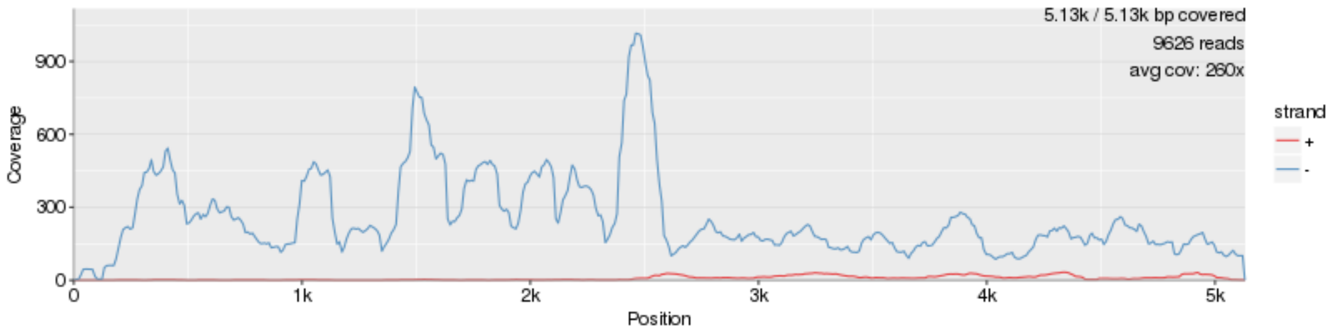

B: Elizabethkingia genomosp. 3 in PT7 (NZ_CP014337.1)

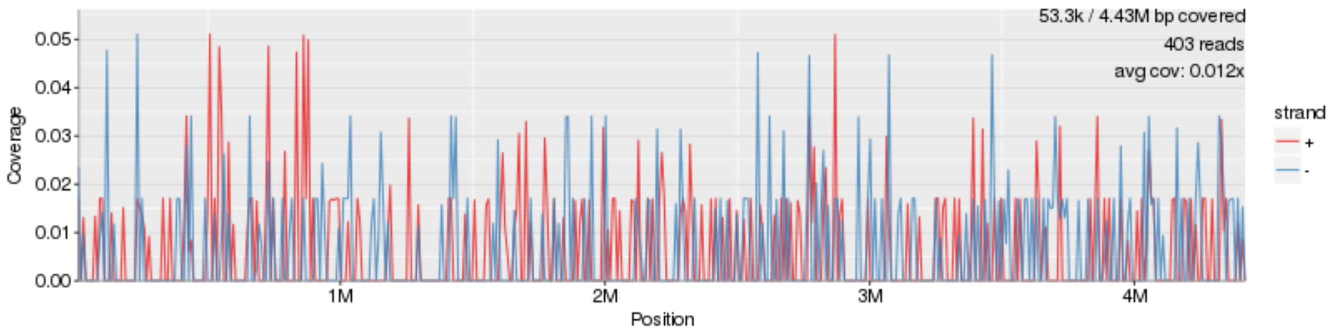

C: Mycobacterium tuberculosis in PT8 (NC_000962.3)

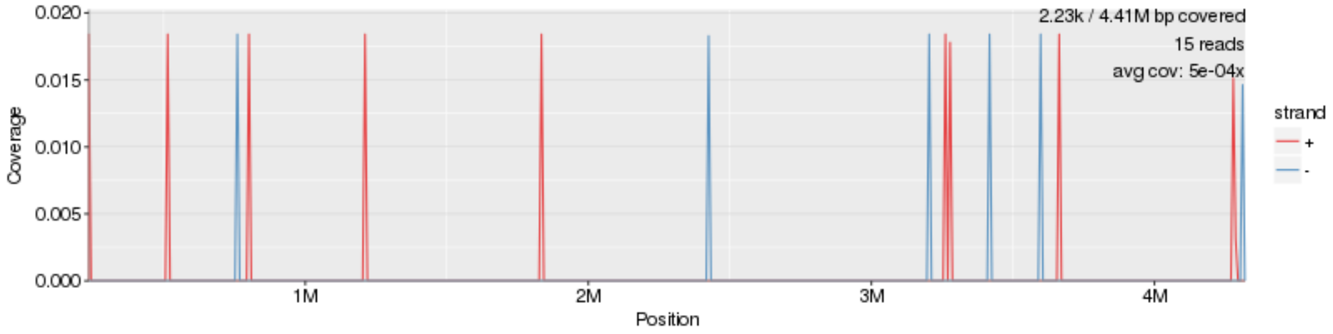

D: Human gammaherpesvirus 4 in PT10 (NC_009334.1)

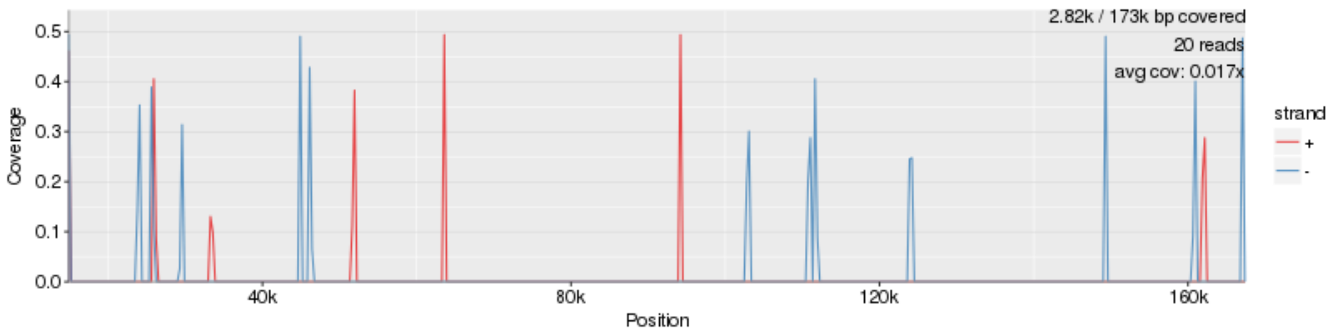

Figure S4: Re-alignment of sequencing samples to specific genomes confirms the observations from the k-mer counts that the reads are pretty randomly distributed across the genomes. The reads were

extracted with krakenuniq-extract-reads, aligned against the reference genome with bowtie2 [10], processed with samtools [11], and visualized with Pavian [12].

Investigation into the likely source of false positives

Without manual investigation it is hard to tell the source of reads that give false identifications. We investigated the false positive identifications in the patient samples listed in Table 4 by extracting the reads and manually searching them against the nt database with NCBI's blastn:

- 122 reads classified as *Clostroides difficile* in PT3: most of these match 16S rRNA sequences in uncultured bacteria.
- 101 reads classified as HCV in PT4: The reads map to many human BAC and fosmid clones and small nucleolar RNAs (snRNA), with the only non-eukaryotic hits corresponding to a recombinant HCV strain that we also detect. Since it seems that this sequence is not part of the human reference genome, KrakenUniq assigned it to the HCV strain. That virus entry actually contains a fragment from a human snRNA, as annotated in the virus sequence entry JF343788.1: "derived from isolate HC-J6; chimeric with small nucleolar RNA U3 fragment".
- 936 reads classified as *Akkermansia muciniphila* in PT5: most matches are to 16S rRNA genes in uncultured bacteria, environmental samples. Very few hits go to *Akkermansia muciniphila*, however we cannot say for certain what the source of this sequence is.
- 63 reads classified as Human betaherpesvirus 5 in PT10: these are matches to the phiX-174 sequence. Even though we have phiX-174 in our database as a contaminant, the algorithm assigned it to HHV5, possibly because the reads seem to be chimeric. Notably, the phiX-174 sequences match to many other sequences in the nt database that are annotated as apicomplexans, flies, flatworms, nematodes, as well as various bacteria.

We also tested whether the reads are of low complexity using dustmasker. Notably, these reads are not of low complexity, probably because we excluded low-complexity k-mers from the database by masking genome sequences.

Table S6: Synthetic constructs (vectors, adapters, primers) and common laboratory and skin contaminants in the pathogen identification sample. Note that the microbes have a high number of unique k-mer per read which indicates that the reads are randomly distributed on their genomes, and that probably the whole genomes are present in the sequenced sample. Data extracted with Pavian [12].

Taxon	synthetic construct (32630)		phiX174 (374840)		*Delftia* (80865)		*Cutibacterium acnes* (1747)		*Escherichia coli* (562)	
Sample	reads	kmers	reads	kmers	reads	kmers	reads	kmers	reads	kmers
**PT1**	48386	1103	91868	9660	8	612	2	330	519	28369
**PT2**	4582	566	4359	2944	19	3190	15	3532	28	1823
**PT3**	52948	1355	12147	4694	238	23769	61	7815	147	5976
**PT4**	31285	1308	22084	7321	0	0	80	7575	160	6306
**PT5**	29022	1849	53401	9405	0	0	461	9918	336	11569
**PT6**	12956	1203	43250	9124	1	66	52	2391	283	9221
**PT7**	268182	1702	13933	5462	301	19070	1430	130773	108	3844
**PT8-S1**	15601	620	7	118	3	191	2	148	0	0
**PT8-S2**	719	529	0	0	0	0	3	237	0	0
**PT9**	6851	951	17562	6378	9	603	220	21028	115	4241
**PT10**	33833	811	17028	5794	0	0	23	2224	115	4389

3. Storing strain genomes with assembly project and sequence accessions

Kraken stores a NCBI taxonomic identifier for each k-mer in its database. This strategy worked well when new taxonomy IDs were assigned to each new microbial strain in GenBank. However, in 2014 the NCBI Taxonomy project stopped assigning new IDs to microbial strains; since then, only novel species get new taxonomy IDs (Federhen, et al., 2014). New microbial genomes, therefore, have the taxonomy

ID of the species, or the taxonomy ID of a strain that was added before 2014. Microbes that have been intensively surveyed, such as Escherichia coli or Salmonella spp., have hundreds of genomes indexed with the same taxonomy ID, and are thus indistinguishable by Kraken. An alternative way of identifying bacterial strains is to use the Bioproject, Biosample and Assembly accession codes [13]. KrakenUniq thus adds new nodes to the taxonomy tree as children of the assigned taxon. A taxonomic node may also be added for each sequence; e.g., specific bacterial chromosomes or plasmids. Those new nodes in the taxonomy tree are given taxonomy IDs starting at 1,000,000,000. Having these extended nodes can help identify specific strains as well as bad database sequences (see **Table 2** and **Table S7**).

The additional information can be useful to detect the source of false positive identifications, too. In the reanalysis of the patient samples [9] with database 'std', *Salmonella enterica* is detected in every sample with up to 233 reads. This species was not detected in the original analysis, and its ubiquity as well as a very low k-mer count hint that it is a false-positive hit or contaminant. If the only available information was the taxonomy ID, the search for the source of these hits would be difficult: There are 349 complete genomes in RefSeq for *Salmonella enterica* (taxonomy ID 28901) and still 23 complete genomes for the strain *Salmonella enterica subsp. enterica serovar Typhimurium* (taxonomy ID 90371). **Table S7** shows a part of the report KrakenUniq generated for PT8. Most of the reads going to *Salmonella enterica* hit one specific plasmid in one strain assembly. With standard Kraken output, neither the number of unique k-mers nor the sequence ID would have been known, and additional investigation such as re-alignment of the reads would have been required.

Reads	Taxon Reads	Kmers	TaxID	Rank	Name
233	0	41	590	genus	Salmonella
233	0	41	28901	species	·Salmonella enterica
232	0	33	59201	subspecies	··Salmonella enterica subsp. Enterica

204	0	19	90371	no rank	···Salmonella enterica subsp. enterica serovar Typhimurium
203	0	8	1000014850	assembly	····GCF_001617585.1 Salmonella enterica subsp. enterica serovar Typhimurium strain=RM9437
203	203	8	1000014852	sequence	·····NZ_CP014577.1 Salmonella enterica subsp. enterica serovar Typhimurium strain RM9437 plasmid pRM9437, complete sequence

Table S7: Part of KrakenUniq output for PT8 [9]. *Salmonella enterica* is likely a false positive identification, and this is indicated by two factors: (1) the unique k-mer count is low. (2) The majority of reads hit a plasmid of one specific strain.

To enable both features, call `krakenuniq-build` with the options `--taxids-for-genomes` and `--taxids-for-sequences`. There is an important drawback to enabling these options: The pseudo-taxonomy IDs - e.g. 1000014850 in **Table S7** - are unique to the database build. Special precautions have to be taken when comparing results from different databases, or when using hierarchical mapping.

4. Integrating viral strain genomes in the database

The RefSeq project curates viral genomes [14], which are included in the default databases of many metagenomics classifiers. RefSeq includes only one reference genome per viral species, and classifiers that use RefSeq (Kraken and others) therefore only consider those genomes. However, there are thousands of viral strain sequences in GenBank, and the chosen reference genome is often an established but old strain. For example, for HIV-1 the reference is a genome assembly from 1999 (AC GCF_000864765.1), and for JC polyomavirus the reference is the strain Mad1 (AC GCF_000863805.1) assembled in 1993. As many viruses exhibit high strain variability, including just the reference genomes in the Kraken database leads to a loss of sensitivity in the detection of strains.

KrakenUniq's database-building script includes the viral strain genomes from the NCBI viral genome resource [14], which maintains a list of 'neighbors' to the viral reference genomes. This list has 112,148 sequences from viral strains, as compared to the 7497 viral genomes in RefSeq (as of October 2017). For example, there are over 2500 additional sequences for HIV-1, and over 640 for JC Polyomavirus. In total, these sequences add 100 million (+33%) novel k-mers to the database with k=31. Based on simulated reads from these viral sequences, 21.2% of the reads would not be classified when searching against a database which includes only the RefSeq viral reference genomes.

5. Hierarchical read classification with multiple databases

KrakenUniq allows using multiple databases hierarchically in order of confidence. In the following example each k-mer is matched first against the HOST, then the PROK, then the EUK_DRAFT database.

```
krakenuniq --db HOST --db PROK --db EUK_DRAFT
```

Note that the KrakenUniq databases need to share the same taxonomy database. If taxonomy nodes are added for genomes or sequences during the database build (parameters --taxids-for-genomes and --taxids-for-sequences), the databases have to be built consecutively using the taxDB file from the previous build.

6. Switching from Kraken to KrakenUniq

KrakenUniq can be used as drop-in replacement to Kraken on a Kraken database. The first run will take longer as KrakenUniq builds its own taxonomy index and counts all k-mers in the database. Note that certain features, such as assembly and sequence identifications, require a full database download and build using KrakenUniq, but the unique k-mer counting works out of the box with a standard Kraken database. Note that `--report-file` on the command line is a required option.

```
krakenuniq --db DB --report-file REPORT_FILE --output KRAKEN_FILE
```

The output file of KrakenUniq is identical to Kraken. The report file has a couple of modifications - namely a header and three additional columns.

- kmers: number of unique k-mers
- dup: average number of times each unique k-mer has been seen
- cov: coverage of the k-mers of the clade in the database

7. New taxonomy database format

KrakenUniq has a new taxonomy format based on code from k-SLAM [15]. The taxDB file lists the taxa in the following form:

```
Taxonomy ID<tab>Parent Taxonomy ID<tab>Rank<tab>Scientific Name
```

KrakenUniq reports all 27 ranks defined in the NCBI taxonomy, instead of just five abbreviated ranks in Kraken ('D' for superkingdom, 'O' for order, 'P' for phylum, 'F' for family, 'G' for genus, 'S' for species). For example, there are species groups and subgroups, subfamilies and varietas.

8. References for the Supplement

1. Heule S, Nunkesser M, Hall A: **HyperLogLog in practice.** 2013**:**683.
2. Ertl O: **New Cardinality Estimation Methods for HyperLogLog Sketches.** *arXiv:170607290* 2017.
3. Flajolet P, Fusy É, Gandouet O, Meunier F: **HyperLogLog: the analysis of a near-optimal cardinality estimation algorithm.** In *AofA: Analysis of Algorithms*; *2007-06-17; Juan les Pins, France*. Discrete Mathematics and Theoretical Computer Science; 2007: 137-156.
4. Whang KY, Vanderzanden BT, Taylor HM: **A Linear-Time Probabilistic Counting Algorithm for Database Applications.** *Acm Transactions on Database Systems* 1990, **15:**208-229.
5. R Core Team: **R: A language and environment for statistical computing.** Vienna, Austria: R Foundation for Statistical Computing; 2017.
6. Wickham H: *ggplot2.* 2009.
7. Menzel P, Ng KL, Krogh A: **Fast and sensitive taxonomic classification for metagenomics with Kaiju.** *Nat Commun* 2016, **7:**11257.
8. McIntyre ABR, Ounit R, Afshinnekoo E, Prill RJ, Henaff E, Alexander N, Minot SS, Danko D, Foox J, Ahsanuddin S, et al: **Comprehensive benchmarking and ensemble approaches for metagenomic classifiers.** *Genome Biol* 2017, **18:**182.
9. Salzberg SL, Breitwieser FP, Kumar A, Hao H, Burger P, Rodriguez FJ, Lim M, Quinones-Hinojosa A, Gallia GL, Tornheim JA, et al: **Next-generation sequencing in neuropathologic diagnosis of infections of the nervous system.** *Neurol Neuroimmunol Neuroinflamm* 2016, **3:**e251.
10. Langmead B, Salzberg SL: **Fast gapped-read alignment with Bowtie 2.** *Nat Methods* 2012, **9:**357-359.
11. Li H, Handsaker B, Wysoker A, Fennell T, Ruan J, Homer N, Marth G, Abecasis G, Durbin R, Genome Project Data Processing S: **The Sequence Alignment/Map format and SAMtools.** *Bioinformatics* 2009, **25:**2078-2079.
12. Breitwieser FP, Salzberg SL: **Pavian: Interactive analysis of metagenomics data for microbiomics and pathogen identification.** *BioRxiv* 2016.
13. Breitwieser FP, Lu J, Salzberg SL: **A review of methods and databases for metagenomic classification and assembly.** *Brief Bioinform* 2017.
14. Brister JR, Ako-Adjei D, Bao Y, Blinkova O: **NCBI viral genomes resource.** *Nucleic Acids Res* 2015, **43:**D571-577.
15. Ainsworth D, Sternberg MJE, Raczy C, Butcher SA: **k-SLAM: accurate and ultra-fast taxonomic classification and gene identification for large metagenomic data sets.** *Nucleic Acids Res* 2017, **45:**1649-1656.
