# Supplementary material for: KrakenUniq: confident and fast metagenomics classification using unique k-mer counts
Source: Genome Biol. 2018 Nov 16;19:198. doi: 10.1186/s13059-018-1568-0 (PMC6238331; doi:10.1186/s13059-018-1568-0)
Supplement: Supplementary file 3 — Table S8, showing the comparison of sequencing depth and k-mer count threshold from Fig. 4. (PDF 93 kb) [file 13059_2018_1568_MOESM3_ESM.pdf]

Table S8: Sequencing depth and k-mer count threshold from Figure 4.

No. of reads	Fraction	Genus		Species	
		Threshold	Recall	Threshold	Recall
1 million	0.03	2555	0.87	3682	0.80
2 million	0.06	4483	0.86	6152	0.81
5 million	0.15	12723	0.87	10459	0.85
10 million	0.3	21896	0.88	21201	0.85
20 million	0.6	43417	0.88	43417	0.84
34.3 million	1	69847	0.89	688842	0.85
